# Protocol for total internal reflection fluorescence microscopy image preprocessing and radial analysis of LFA-1 in T cell immune synapses

**DOI:** 10.1016/j.xpro.2026.104636

**Published:** 2026-06-17

**Authors:** Lewis Murugu, Johan Henriksson, Hudson Pace, Marta Bally, Lena Svensson

**Affiliations:** 1 Department of Molecular Biology, Umeå University, 90187 Umeå, Sweden; 2 Department of Clinical Microbiology, Umeå University, 90187 Umeå, Sweden; 3 Umeå Centre for Microbial Research (UCMR), Umeå University, 90187 Umeå, Sweden; 4 Science for Life Laboratory (SciLifeLab), Umeå University, Umeå, Sweden

**Keywords:** Bioinformatics, Cell Biology, Immunology, Microscopy, Molecular Biology

## Abstract

Here, we present a protocol for total internal reflection fluorescence (TIRF) microscopy image preprocessing and radial analysis of lymphocyte function-associated antigen-1 (LFA-1) in T cell immune synapses. We describe steps for combining customizable Fiji/ImageJ macros for image background subtraction and segmentation with flexible R-based scripts for peripheral supramolecular activation cluster (pSMAC) center identification, center-based cropping, and radial analysis of synaptic LFA-1 in T cells. This workflow supports established analyses, including radial averaging/spinning and fluorescence intensity measurements across the synapse.

## Highlights

• Steps for immune synapse image background subtraction and ROI segmentation
• Instructions for synapse center detection and aligned image cropping
• Steps for LFA-1 fluorescence quantification and radial profiling
• Guidance on F-actin measurement and radial averaging analysis

## Abstract (editor highlights)

Publisher’s note: Undertaking any experimental protocol requires adherence to local institutional guidelines for laboratory safety and ethics.

## Graphical abstract

**Figure:**
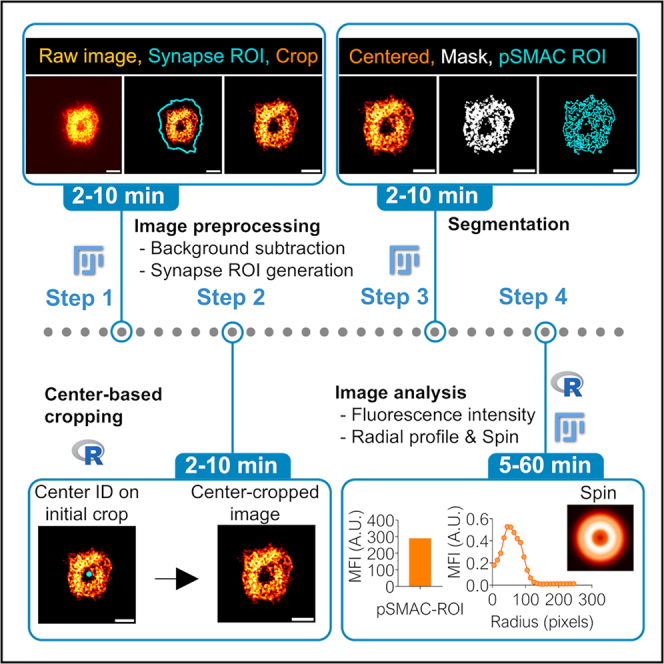

## Before you begin

**Figure 1:**
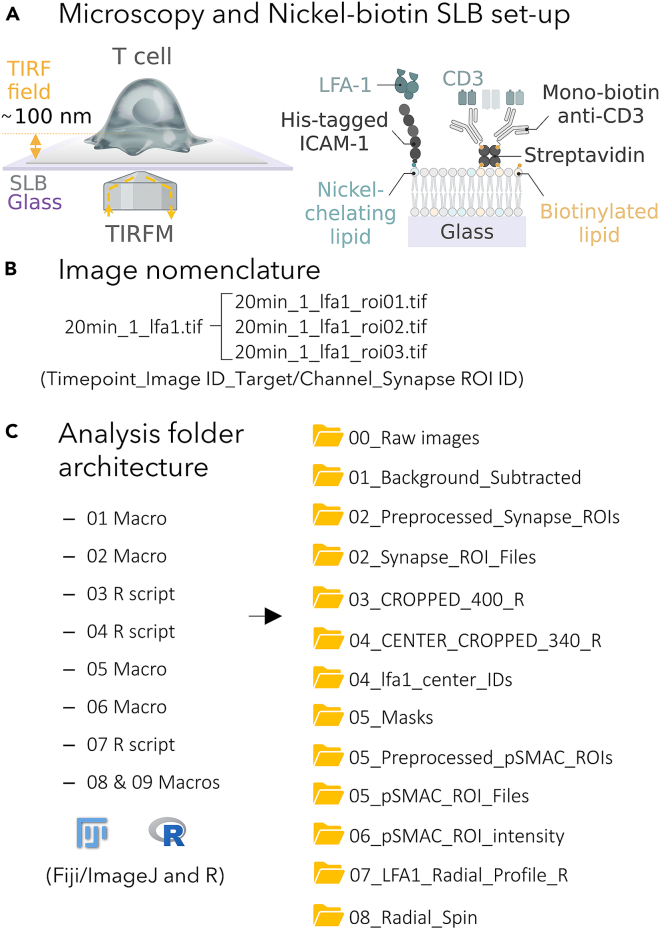
Microscopy set-up and image analysis workflow (A) Schematic of the supported lipid bilayer (SLB)–total internal reflection fluorescence (TIRF) microscopy setup used to image T cell immune synapses. (B) Image nomenclature scheme. Image filenames encode the synapse maturation time point, image identifier, acquired target or fluorescence channel, and synapse region-of-interest (ROI) identifier following initial image cropping. (C) Folder structure and output directory organization generated after image analysis.

Lymphocyte function–associated antigen 1 (LFA-1) is a β2 integrin that organizes into a distinct spatial domain at the immunological synapse formed between T cells and antigen-presenting cells (APCs). Similarly, T cells can form immune synapses with reductionist planar lipid membranes, called supported lipid bilayers (SLBs), that are ligand-functionalized to mimic APC surfaces. In mature synapses, LFA-1 accumulates in the peripheral supramolecular activation cluster (pSMAC), where it forms a prominent adhesive ring and acts as a key integrin receptor.^1^^,^^2^^,^^3^^,^^4^^,^^5^^,^^6^ Its organized distribution within the synapse enables LFA-1 to mediate firm adhesion, regulate ligand engagement, and coordinate cytoskeletal dynamics, collectively stabilizing and shaping synapse architecture.^1^^,^^2^^,^^6^^,^^7^ To directly visualize synaptic spatial features, T cells interacting with functionalized SLBs are commonly imaged using total internal reflection fluorescence (TIRF) microscopy. As shown in Figure 1A, this technique provides high-resolution views by selectively illuminating the ∼100–200 nm interface at the T cell–SLB contact, where synaptic structures polarize. However, despite its widespread use, access to flexible, batch-compatible image-processing and analysis tools remains limited.

Here, we present a streamlined workflow for batch analysis of TIRF microscopy images of T cell immune synapses. To demonstrate its application, we use images of human CD4^+^ T cells forming immunological synapses on a nickel–biotin SLB platform. In this system, as illustrated in Figure 1A, the SLB is formed on a glass surface and functionalized with His-tagged ICAM-1 via nickel-chelating lipids, together with monobiotinylated anti-CD3 antibody (OKT3) tethered through streptavidin to biotinylated lipids.^8^^,^^9^ T cells are allowed to form synapses and are then fixed at defined time points (5, 10, 20, and 30 minutes) to capture different stages of synapse formation, followed by staining for key synaptic markers (CD3, LFA-1, and F-actin) using fluorescently labeled antibodies and phalloidin.

Our image processing and analysis workflow combines ImageJ macros for background subtraction and synapse region-of-interest (ROI) segmentation with R-based scripts for automated initial image cropping, detection of the pSMAC center from LFA-1 fluorescence, and subsequent center-aligned cropping of all co-acquired channels. We also describe ImageJ macros for segmentation of LFA-1-enriched pSMAC regions and ROI-based fluorescence intensity quantification, together with an R-based workflow for radial analysis of LFA-1 distribution within the pSMAC.

Finally, the center-cropped images generated earlier are processed through radial spinning/averaging in ImageJ,^10^^,^^11^^,^^12^ an established method for quantifying protein-distribution dynamics across the central (cSMAC), peripheral (pSMAC), and distal (dSMAC) supramolecular activation cluster regions of the immunological synapse.

### Innovation

Analysis of LFA-1 from TIRF microscopy images of T cell immune synapses is often hindered by the lack of standardized, automated, and accessible tools for batch preprocessing, segmentation, pSMAC-center detection, and spatial quantification across large multi-image datasets. Existing approaches rely heavily on custom methods described succinctly within specific experimental contexts in primary research articles, making them difficult to reproduce, benchmark, or adapt across different imaging setups or analysis needs.

This protocol addresses these limitations by providing an integrated and automated workflow that streamlines image preprocessing, synapse ROI segmentation, and reliable pSMAC–center detection using combined ImageJ and R-based tools. We further demonstrate that the framework supports multiple complementary analysis modes, including radial profiling of LFA-1 and ROI-based fluorescence quantification, and can be readily extended to additional synaptic markers such as F-actin. Importantly, the center-aligned outputs are fully compatible with established radial spinning methods, facilitating standardized spatial characterization across central, peripheral, and distal SMAC regions.

### Acquire immune synapse images

**Timing: Variable**

1. Acquire immune synapse images using TIRF microscopy.
2. Export the images in TIFF format for downstream analysis.

***Note:*** Images used in this protocol were acquired using a Zeiss Axio inverted microscope equipped with a Zeiss alpha Plan-Apochromat 100×/1.46 Oil DIC M27 objective, 405-, 445-, 488-, 561-, and 640-nm laser lines and a Hamamatsu C11440-22CU/ORCA-flash 4.0 digital camera. Image acquisition was performed using 3i SlideBook2023 software.

### Download and install R, RStudio, and Fiji/ImageJ

**Timing: 5 min**

3. Download and install the latest versions of R, RStudio, and Fiji/ImageJ from their official websites.
  a. Download R from http://www.r-project.org/ and RStudio from https://posit.co/downloads/.
  b. Download Fiji/ImageJ from https://imagej.net/ij/.
  c. Follow the installation instructions provided on each website to ensure proper setup on Windows, macOS or Linux.

***Note:*** The analyses in this protocol were performed using R (v4.5.1) and ImageJ (v1.5r) running on Windows 11.

### Download required macros and R scripts

**Timing: 2 min**

4. Download the ImageJ macros and R scripts needed for analysis at Zenodo: https://doi.org/10.5281/zenodo.17913303.

### Download required R packages

**Timing: 1–3 min**

5. Download, install and load the required packages in RStudio.
  a. Open RStudio, install and load these packages.

## CRAN packages

install.packages(c(

 "magick",

 "RImageJROI",

 "ggplot2",

 "dplyr",

 "tidyr",

 "stringr"

))

library(magick)

library(RImageJROI)

library(ggplot2)

library(dplyr)

library(tidyr)

library(stringr)

library(tools) # base R package

## Bioconductor package

if (!requireNamespace("BiocManager", quietly = TRUE))

 
install.packages("BiocManager")

BiocManager::install("EBImage")

library(EBImage)

### Image nomenclature and analysis folder structure

**Timing: N/A**

Images used in this protocol follow a consistent naming convention that is preserved across all processing steps and relied upon by downstream analyses. The example dataset includes three synaptic proteins—CD3, LFA-1, and F-actin—as well as the corresponding Differential interference contrast (DIC) images. Edit the image nomenclature to match the specific time points and targets stained in your experiment.

The protocol further assumes a predefined project folder structure that is maintained throughout all analysis steps. A single project (parent/base) directory is used to store raw data and all downstream outputs. Subfolders generated by ImageJ macros and R scripts are created automatically within this directory.

An overview of the image-naming scheme and analysis folder structure is shown in Figures 1B and 1C.

## Key resources table

**Table:** 

REAGENT or RESOURCE	SOURCE	IDENTIFIER
**Deposited data**		

Raw TIRF microscopy images	This paper	https://doi.org/10.5281/zenodo.17913303
R scripts	This paper	https://doi.org/10.5281/zenodo.17913303
ImageJ macros	This paper	https://doi.org/10.5281/zenodo.17913303
Analysis output	This paper	https://doi.org/10.5281/zenodo.17913303

**Software and algorithms**		

R	The R Project for Statistical Computing	http://www.r-project.org/
RStudio Desktop	RStudio	https://posit.co/downloads/
ImageJ software	Schneider et al.^13^	https://imagej.net/ij/
magick	Ooms^14^	https://cran.r-project.org/package=magick
RImageJROI	Mainspeizer et al.^15^	https://cran.r-project.org/package=RImageJROI
EBImage	Pau^16^	https://bioconductor.org/packages/EBImage
ggplot2	Wickham et al.^17^	RRID:SCR_014601; https://cran.r-project.org/package=ggplot2
dplyr	Wickham et al.^18^	RRID:SCR_016708; https://cran.r-project.org/package=dplyr
tidyr	Wickham et al.^19^	https://cran.r-project.org/package=tidyr
stringr	Wickham et al.^20^	RRID:SCR_016222; https://cran.r-project.org/package=stringr
tools	R base package	Included with R

## Step-by-step method details

### Image preprocessing: Verification of image nomenclature and spatial calibration

**Timing: Variable**

Image preprocessing begins with verification of image naming and spatial calibration consistency with metadata to support accurate downstream quantitative analysis.

1. Verify that all image files follow the naming convention described in this protocol.
2. Open images and verify spatial calibration.
  a. Launch ImageJ and open an image using the Bio-Formats Importer (Plugins > Bio-Formats > Bio-Formats Importer).
  b. Navigate to the directory containing the images.
  c. Select an image file and click Open.
  d. In the Bio-Formats Import Options window, select Display Metadata.
  e. Record the pixel size (e.g., μm/pixel) from the Original Metadata window.
  f. To verify spatial calibration, select Analyze > Set Scale.
  g. Confirm that the Distance in pixels, Known distance, and Unit of length match the metadata values.

***Note:*** Incorrect file naming or missing/incorrect spatial calibration will cause downstream scripts and macros to fail. (Troubleshooting 1 and Troubleshooting 2).

### Image preprocessing: Background subtraction

**Timing: 1–2 min**

This step performs batch background subtraction of TIRF microscopy images using the ImageJ macro “01_Macro for background subtraction.” Background subtraction is performed using the rolling ball algorithm in ImageJ, which estimates local background intensity within a defined radius and subtracts it from the image. The radius parameter (in pixels) determines the spatial scale of background estimation: larger values remove broad, low-frequency intensity variations, whereas smaller values may lead to overcorrection and loss of local signal.

***Note:*** A rolling ball radius of 50 pixels was used for all channels, as empirically determined to preserve synaptic features while removing diffuse background. This value may require adjustment depending on image signal intensity, background levels and feature size. (Troubleshooting 3).

**Figure 2:**
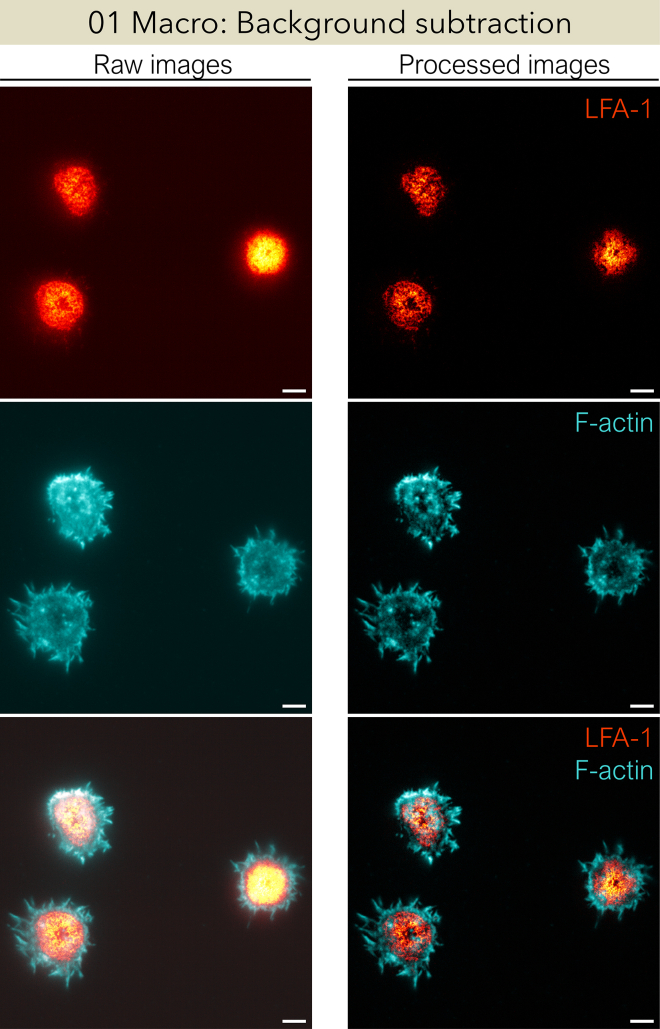
Background subtraction of TIRF microscopy images Representative images shown before and after background subtraction. Scale bar: 5 μm.

***Note:*** The macro automatically generates a nested output folder named “01_Background_Subtracted” within the project/base directory and saves the background-subtracted images there. Representative images before and after background subtraction are shown in Figure 2.

***Note:*** Macros and R scripts used in this protocol are prefixed with numerical identifiers (e.g., “01”) to indicate the order in which they should be executed.

3. Open ImageJ.
4. Load the background subtraction macro.
  a. To load the macro, go to File > Open, navigate to the folder containing the ImageJ macros, select “01_Macro for background subtraction”, and click Open.

Alternatively, drag the “01_Macro for background subtraction” macro file directly into the open ImageJ window.

5. If needed, adjust channel names in the macro to suit your specific channels or synaptic targets.
6. Click Run in the macro window.
7. Select the input directory containing the raw (unprocessed) TIRF microscopy images.
8. Select the “BASE” output directory, which serves as the project (parent) folder for all processed output.

**CRITICAL:** Use the same project directory for all subsequent analysis steps to preserve the automated folder structure and enable traceability to earlier processing stages.

9. Specify the rolling ball radius (in pixels) for each channel (e.g., CD3, LFA-1, F-actin).

***Note:*** The default background subtraction radius is 50 pixels for each channel. This value can be adjusted if required. (Troubleshooting 1 and Troubleshooting 3).

### Image preprocessing: Synapse ROI generation

**Timing: 1–2 min**

This step segments outer synapse regions of interest (ROIs) by thresholding the dSMAC signal using the ImageJ macro “02_Macro for segmenting outer synapse ROIs.” Segmentation is performed on the F-actin channel following preprocessing steps including contrast enhancement (0.35% saturation) and Gaussian smoothing (sigma = 1.5) to reduce noise. An automated thresholding approach (Li method) is then applied to generate a binary mask, which is refined by hole filling to ensure continuous regions. ROIs are subsequently defined using particle analysis based on size and circularity constraints.

***Note:*** The dSMAC (F-actin) signal is used to define the outer synapse ROI because it corresponds to the outermost zone of the immune synapse. This approach is particularly useful when interference reflection microscopy (IRM) imaging is not available to delineate the T cell–SLB contact area.

***Note:*** This macro generates two output folders within the BASE directory: “02_Preprocessed_Synapse_ROIs,” which contains thresholded mask images, and “02_Synapse_ROI_Files,” which contains the corresponding ROI files.

**Figure 3:**
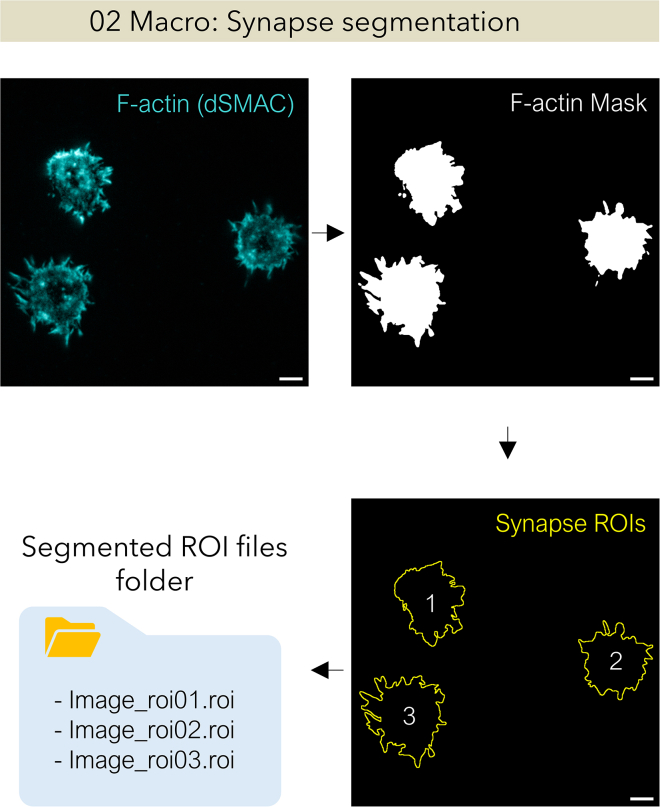
Synapse region-of-interest (ROI) segmentation A representative example illustrating immune synapse ROI definition based on F-actin fluorescence. Scale bar: 5 μm.

***Note:*** Illustrative thresholded binary masks and segmented F-actin images are shown in Figure 3.

10. Load and run the “02_Macro for segmenting outer synapse ROIs” macro in ImageJ.
11. Specify the input folder as “01_Background_Subtracted,” which contains the background-subtracted images, including the F-actin channel.

**CRITICAL:** Inspect the thresholded F-actin (dSMAC) masks to confirm accurate segmentation of the outer synapse region before proceeding to downstream analysis.

### Image preprocessing: Initial synapse cropping

**Timing: 1–2 min**

Individual synapses are cropped from background-subtracted images using synapse ROIs generated from F-actin (dSMAC) segmentation. Cropping is performed using the R script “03_R script for cropping images based on roi files.”

**Figure 4:**
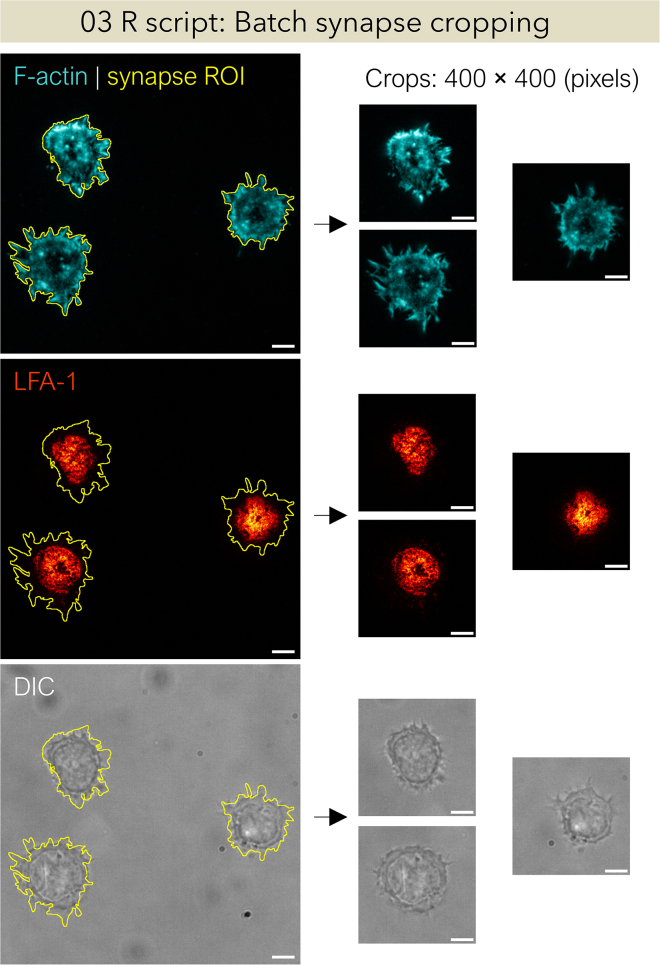
Automated batch immune synapse ROI cropping Example images illustrating initial square cropping (400 × 400 pixels) generated relative to the synapse ROI boundary. Scale bar: 5 μm.

***Note:*** The R script crops square ROIs (400 × 400 pixels) from the original 1024 × 1024-pixel images for all acquired channels (DIC, CD3, LFA-1, and F-actin) and saves the cropped images to a specified output folder within the BASE directory. A representative example of synapse cropping is shown in Figure 4.

***Note:*** The ROI size (400 × 400 pixels) is user-defined within the script and can be adjusted to accommodate differences in pixel size (μm/pixel), magnification, or binning across imaging systems.

12. In RStudio, open the image cropping R script.
  a. Go to File > Open File and locate the “03_R script for cropping images based on roi files” script.
13. Edit the user-defined paths.
  a. Specify the image directory containing background-subtracted images (01_Background_Subtracted).
  b. Define the directory containing synapse ROI files (02_Synapse_ROI_Files).
  c. Set the BASE (parent) directory where the output folder “03_CROPPED_400_R” will be created.

***Note:*** The desired output directory path (out_dir) is user-specified in the script. The output folder does not need to be created manually; the R script will generate it automatically.

**CRITICAL:** Ensure that the paths to the input image folder (01_Background_Subtracted) and ROI folder (02_Synapse_ROI_Files) are identical to those used in previous steps and are maintained consistently in all subsequent steps to preserve the analysis folder structure. (Troubleshooting 4).

**CRITICAL:** Ensure that the channel names in the R script match your image nomenclature scheme.

# -----------------------------

# Load required packages

# -----------------------------

library(magick)

library(RImageJROI)

# -----------------------------

# USER-DEFINED PATHS (edit these)

# -----------------------------

img_dir <- "path/to/01_Background_Subtracted" # Background-subtracted images

roi_dir <- "path/to/02_Synapse_ROI_Files" # Synapse ROI files

out_dir <- "path/to/03_CROPPED_400_R" # Output parent directory

dir.create(out_dir, showWarnings = FALSE, recursive = TRUE)

14. Run the script.

### Image preprocessing: Image quality control

**Timing: Variable**

Images that are unsuitable for analysis (e.g., photobleached, out of focus, or containing imaging artifacts) are manually identified and excluded from the analysis pipeline.

15. Open all images in ImageJ in batches and visually inspect them to identify images that are unsuitable for analysis due to photobleaching, poor focus, or other imaging artifacts.
16. Record the file names of the unfitting images in a text file and save this file in the parent directory.
17. Open and run the macro, “Macro for removing images in a folder by name.”
18. When prompted, select the input folder (03_CROPPED_400_R) and the text file containing the names of images to be removed.

***Note:*** The macro generates a subfolder named “Removed” within the selected input folder and moves all excluded images into this folder.

### Image preprocessing: pSMAC center-based immune synapse cropping

**Timing: 1–2 min**

In this step, the immune synapse center is identified using the pSMAC (LFA-1) channel and images cropped based on this coordinate using the R script “04_0_R script for batch pSMAC center id and center cropping coacquired channels.” Since LFA-1 forms a concentric ring at the immune synapse, its spatial distribution provides a robust reference for center identification.

To identify the synapse center, ROI-cropped LFA-1 images are smoothed and thresholded (Otsu method) to isolate LFA-1–positive signal. The resulting mask is filled to generate a contiguous synapse region, and the largest region is selected. The synapse center is defined as the geometric centroid of this region. A square crop (340 × 340 pixels) centered on this coordinate is then applied identically to all channels (CD3, LFA-1, F-actin, and DIC).

***Note:*** The default ROI size used here (340 × 340 pixels) is user-defined within the script and can be modified as needed.

***Note:*** Synapses that cannot be cropped without edge truncation are excluded from further analysis. Depending on the number of affected image crops, this may result in data loss. (Troubleshooting 5).

**Figure 5:**
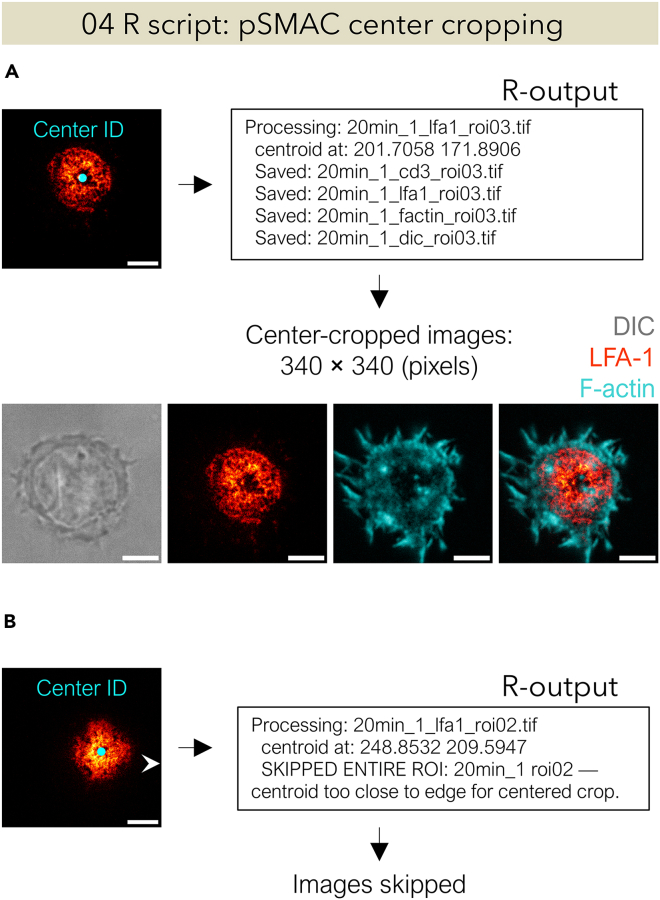
Immune synapse center-based image cropping (A) LFA-1 fluorescence image used to determine the pSMAC center, which is then applied by an R script to batch-crop all corresponding channels to 340 × 340 pixels. (B) Example illustrating a synapse located too close to the edge of the acquired image, where center-based cropping cannot be performed due to insufficient image area and the synapse is therefore excluded from cropping. The white arrow indicates the boundary of the acquisition field. Scale bar: 5 μm.

***Note:*** Selected images illustrating pSMAC center–based cropping are shown in Figure 5.

19. Open the script, “04_0_R script for batch pSMAC center id and center cropping coacquired channels” in RStudio.
20. Edit the user-defined paths.
  a. Specify the image directory containing the initial uncentered crop images (03_CROPPED_400_R).
  b. Set the BASE (parent) directory where the output folder “04_CENTER_CROPPED_340_R” will be created.

**CRITICAL:** It is recommended to keep the directory organization consistent with previous analysis steps. (Troubleshooting 4).

21. Run the script.
22. To visualize the identified synapse center coordinates, run the script “04_1_R script for pSMACs center ID labeling” in RStudio using the same procedure as above. However, edit the user-defined paths.
  a. Specify the image directory containing either the initial uncentered crop images (03_CROPPED_400_R) or the center-cropped images (04_CENTER_CROPPED_340_R), depending on which stage of processing you wish to visualize.
  b. Set the base (parent) directory in which the output folder “04_lfa1_center_IDs” will be created.
  c. Run the script.

### Image preprocessing: pSMAC ROI segmentation

**Timing: 1–2 min**

Center-cropped pSMAC (LFA-1) images are thresholded to generate binary masks, which are used to segment pSMAC ROIs. Thresholding is performed using an automated approach in ImageJ following background subtraction (rolling ball radius = 15) and smoothing, with the “Mean” thresholding method applied to identify LFA-1–positive regions. The resulting masks are converted into selections, added to the ROI Manager, and saved as ROI files for downstream analysis.

**Figure 6:**
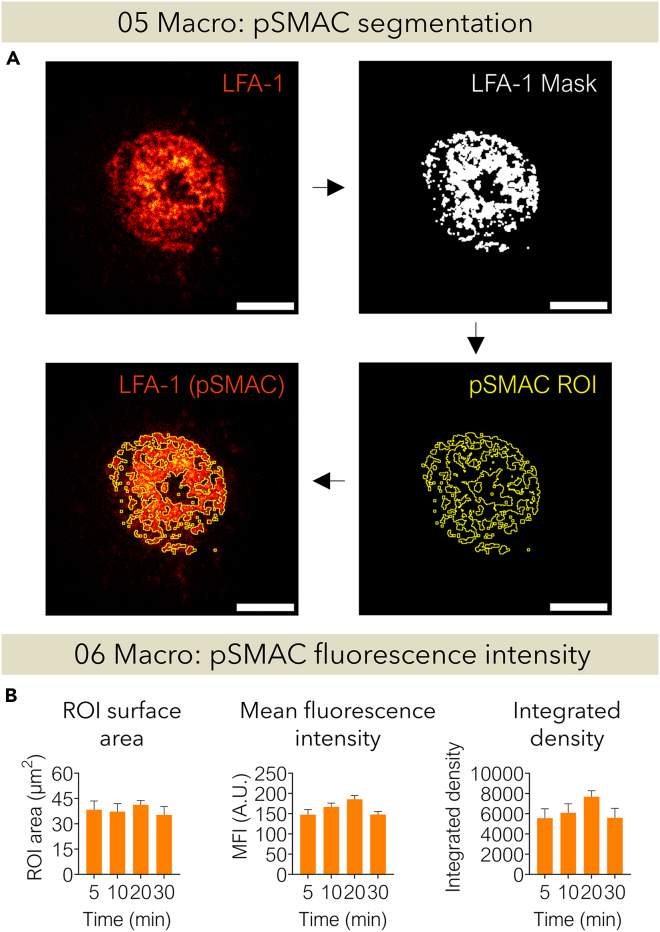
Segmentation and fluorescence quantification of LFA-1 in the pSMAC (A) Representative LFA-1 image illustrating threshold-based segmentation to generate a pSMAC mask and corresponding pSMAC region of interest (ROI). (B) Quantification of pSMAC ROI area, mean fluorescence intensity, and integrated density at different stages of immune synapse formation. Data are shown as mean ± SEM (n > 18). Scale bar: 5 μm.

***Note:*** An illustration of pSMAC ROI segmentation is shown in Figure 6A.

23. In ImageJ, load the macro, “05_Macro for pSMAC thresholding and ROI segmentation.”
24. Run the macro.
25. Select the input directory containing the center-cropped LFA-1 images (04_CENTER_CROPPED_340_R).

***Note:*** The macro automatically identifies the parent directory of the selected input folder, processes the images and generates the output folder “05_pSMAC_ROI_Files” (segmented pSMAC ROIs), “05_Preprocessed_pSMAC_ROIs” (preprocessed LFA-1 images prior to thresholding), and “05_Masks” (binary masks of pSMAC regions) within the same parent directory.

**CRITICAL:** Ensure that the selected input folder is located within the same base (parent) directory used in previous steps to preserve the analysis folder architecture.

26. Inspect the generated binary masks to confirm that the segmented pSMAC ROIs accurately correspond to the LFA-1 signal in the original images before proceeding to quantitative analysis.

### Image analysis: Batch pSMAC ROI fluorescence intensity quantification

**Timing: 1–2 min**

In this step, pSMAC ROIs generated in the previous section are used to quantify LFA-1 fluorescence intensity within the pSMAC.

27. Open the macro, “06_Macro for batch pSMAC fluorescence intensity profile measurement” in ImageJ.
28. Run the macro.
29. Select the input folder containing the LFA-1 images (04_CENTER_CROPPED_340_R), the “05_pSMAC_ROI_Files” folder containing the pSMAC ROIs, and the parent directory in which the output folder “06_pSMAC_ROI_intensity” will be created.

**CRITICAL:** Ensure that all selected input folders are located within the same base (parent) directory used in previous steps to preserve the analysis folder architecture.

***Note:*** The macro generates a CSV file (ROI_Intensity_Results.csv) in the output folder that reports pSMAC ROI area, mean fluorescence intensity, integrated density (mean intensity × ROI area), and raw integrated density (sum of pixel intensities within the ROI) for each pSMAC ROI. Quantification of pSMAC ROI area and LFA-1 fluorescence intensity across synapse maturation time points is shown in Figure 6B. All three parameters increase from 5 min, peak at 20 min, and decline by 30 min, consistent with the formation and subsequent destabilization of a mature immunological synapse.

### Image analysis: Batch LFA-1 radial analysis

**Timing: 1–2 min**

Radial profiles are plotted as mean fluorescence intensity (y-axis) as a function of distance from the synapse center (x-axis), where each data point represents the average intensity within a concentric radial bin.

30. In RStudio, open the script, “07_R script for radial analysis.”
31. Edit the user-defined paths.
  a. Specify the image directory containing the centered, cropped LFA-1 images (04_CENTER_CROPPED_340_R).
  b. Set the base (parent) directory in which the output folder “07_LFA1_Radial_Profile_R” will be created. (Troubleshooting 4).
32. Confirm that LFA-1 image filenames include a timepoint identifier, as the script assigns timepoints based on filename annotations (e.g., 5min, 10min, 20min, or 30min). Modify the script and/or image filenames if required.
33. Run the script to perform batch radial analysis.

**Figure 7:**
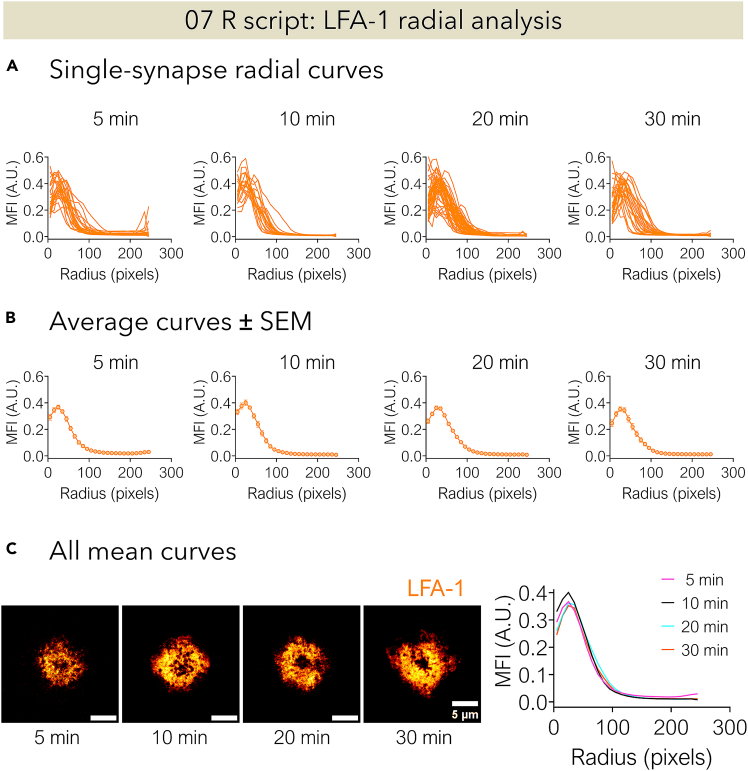
Radial analysis of LFA-1 distribution at the immunological synapse (A) Radial intensity profiles for individual synapses. (B) Mean profiles across cells. (C) Representative images showing LFA-1 expression and an overlay of mean profiles from all synapse maturation time points. Data are shown as mean ± SEM (n > 18).

***Note:*** The script automatically normalizes LFA-1 fluorescence images, segments the cell footprint to define the synapse center, and computes radial LFA-1 intensity profiles using concentric annular bins (10-pixel bin width, extending up to 300 pixels from the center). Separate output folders are generated for each synapse maturation time point (5, 10, 20, and 30 min), containing CSV files of radial profile values, plots of individual synapse profiles, and plots showing the mean LFA-1 radial intensity profile ± SEM across cells (see Figure 7), enabling quantitative comparison of time-dependent changes in synapse organization.

***Note:*** Radial analysis enables robust quantification of the overall spatial distribution of synaptic components and facilitates comparison across multiple cells by reducing spatial information to a function of distance from the synapse center. While image acquisition is typically focused on well-formed synapses with approximately concentric organization, variability in synapse geometry and local molecular distribution can still occur, particularly under conditions where synapse morphology may be altered. As a result, this approach may obscure angular heterogeneity or morphological features such as variations in synapse shape or cytoskeletal organization. Individual synapse inspection and complementary analyses may be informative where such features are of interest.

### Image analysis: Batch F-actin ROI fluorescence intensity quantification and radial analysis

**Timing: 1–2 min**

**Figure 8:**
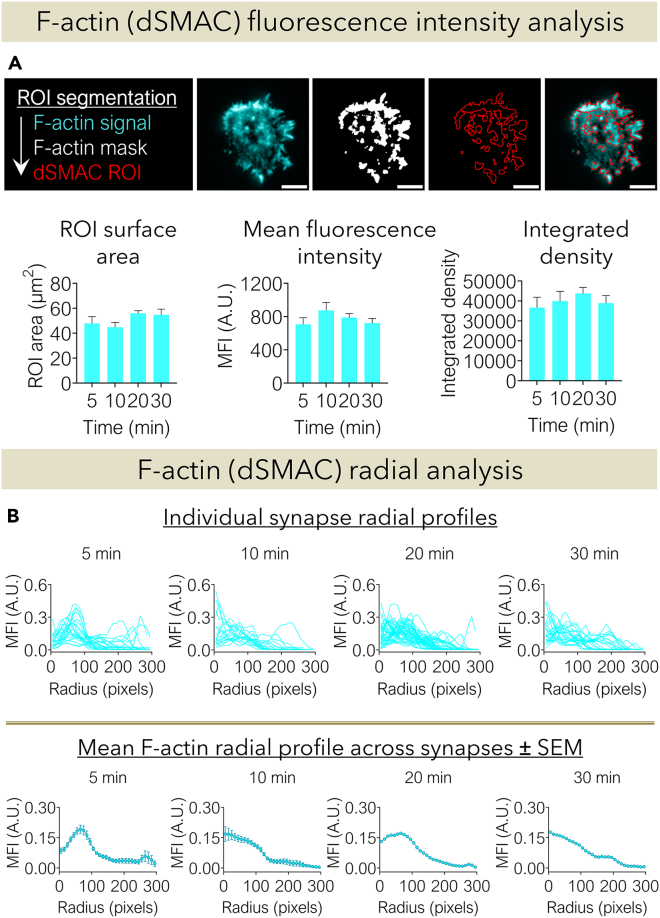
F-actin fluorescence intensity quantification and radial analysis (A) Schematic showing segmentation of F-actin ROIs and intensity measurements. (B) Radial analysis of time-dependent F-actin dynamics at the synapse Data are shown as mean ± SEM (n > 18). Scale bar: 5 μm.

In addition to LFA-1, the ROI-based fluorescence intensity quantification and radial analysis workflows can be applied to other synaptic markers with concentric spatial organization, such as F-actin (dSMAC) (see Figure 8).

34. Segment F-actin ROIs using the ImageJ macro “05_Macro for pSMAC thresholding and ROI segmentation” by following the same steps described above.
35. Edit the macro to replace all instances of the channel name lfa1 with factin, so that the macro processes F-actin images within the folder 04_CENTER_CROPPED_340_R.
36. Quantify F-actin ROI fluorescence intensity using the macro “06_Macro for batch pSMAC fluorescence intensity profile measurement”, replacing all instances of lfa1 with factin.
37. To perform radial analysis of F-actin, edit the R script “07_R script for radial analysis” by replacing all instances of lfa1 with factin.

**CRITICAL:** As done for LFA-1 analysis, maintain the same parent directory structure.

### Image analysis: Radial averaging (radial spinning)

**Timing: Variable**

This section describes the application of the image preprocessing workflow outlined in this protocol to a method for analyzing the average spatial organization of proteins at the immunological synapse, commonly referred to as radial spinning or radial averaging. In this approach, center-aligned TIRF microscopy images are rotated about the synapse center in 1° increments to generate 360 rotated images. These images are then averaged using Z-projection to produce a radially averaged image, from which a line profile is extracted to generate an average radial intensity profile across the synapse.^10^^,^^11^^,^^12^

***Note:*** The macros used in this section are adapted from literature illustrating radial averaging.^11^^,^^12^

**Figure 9:**
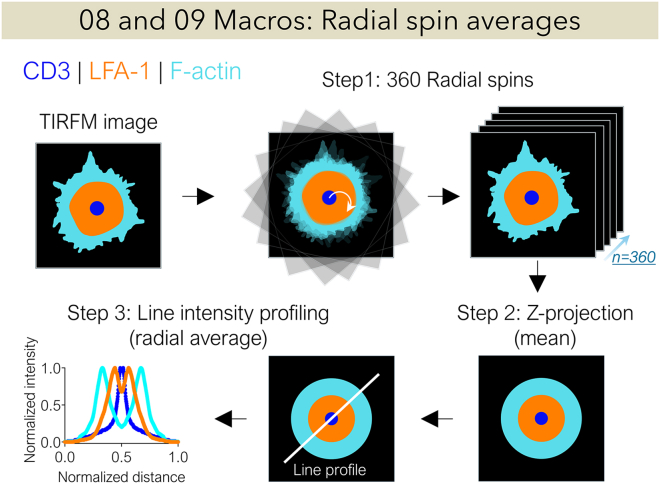
Radial spinning analysis Workflow illustrating radial spinning, Z-projection, and line intensity profiling used to generate radial average intensity curves.

**Figure 10:**
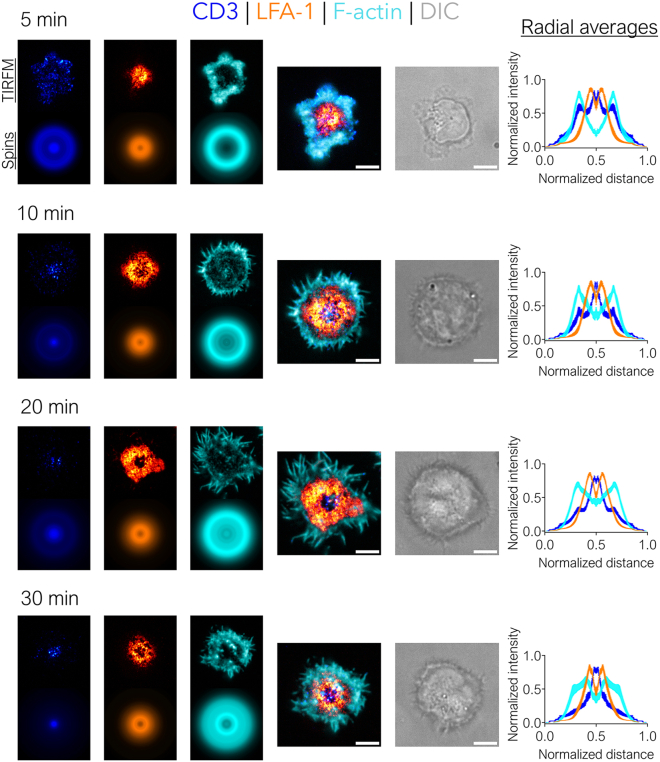
Radial spinning analysis of immune synapse components Schematic illustrating radial averaging of CD3 (cSMAC), LFA-1 (pSMAC), and F-actin (dSMAC) distributions at different time points of immune synapse maturation. Data are shown as mean ± SEM from >18 synapses per condition. Scale bar: 5 μm.

***Note:*** A schematic illustrating radial averaging of CD3, LFA-1, and F-actin is shown in Figure 9. Temporal dynamics of these proteins during different stages of immune synapse formation are shown in Figure 10.

38. Open the ImageJ macro “08_Macro for radial spinning images and skips DICs.”
39. Run the macro.
40. When prompted, select the input folder “04_CENTER_CROPPED_340_R” containing all center-cropped images.
41. Choose the output (parent) directory in which the folder “08_Radial_Spin” will be created.

***Note:*** Radial spinning is computationally demanding and may take several hours to complete, particularly when processing large numbers of images.

42. After radial spinning is complete, create channel-specific subfolders within “08_Radial_Spin” (e.g., CD3, LFA-1, F-actin), and move the corresponding radial spin images into their respective folders.
43. Open and run the ImageJ macro “09_Macro for line profiling radial spin images.”
44. When prompted, select the input channel-specific folders containing the radial spin images.

***Note:*** The line profiling macro generates CSV files containing radial line profiles, organized by timepoint within each channel-specific folder.

## Expected outcomes

This protocol enables a sequential, semi-automated workflow for preprocessing and analyzing TIRF microscopy images of T cell immune synapses formed on ligand-functionalized supported lipid bilayers (SLBs), with a particular focus on tracking the expression and spatial organization of LFA-1. With minor modifications, the workflow can be readily adapted to analyze additional synaptic markers.

Using the provided ImageJ macros and R scripts, users will generate batch background-subtracted images (Figure 2) and define immune synapse regions of interest (ROIs) based on F-actin enrichment, allowing automated segmentation of synapses across datasets (Figures 3 and 4). The protocol further enables identification of synapse centers using LFA-1 signal, followed by batch cropping of all acquired fluorescence channels relative to the synapse center (Figure 5).

From these centered images, users will obtain automated segmentation of the LFA-1–enriched pSMAC, facilitating ROI-based fluorescence intensity quantification across multiple time points of immune synapse formation (Figure 6). The aligned datasets also enable quantitative radial intensity profiling of LFA-1 distributions (Figure 7), an approach that can be directly extended to other synaptic components such as F-actin (Figure 8).

Finally, this protocol allows implementation of radial averaging (“radial spinning”), an analysis tool used to quantify the spatiotemporal organization of synaptic proteins during immune synapse maturation.^10^^,^^11^^,^^12^ Representative outputs of this analysis are shown in (Figure 10). Collectively, the workflow yields standardized, center-aligned synapse datasets suitable for robust comparative analysis of immune synapse architecture and dynamics.

## Limitations

Although this protocol enables a substantial degree of automation, the individual analysis steps must be executed separately and sequentially; a single, fully integrated “one-code” workflow is not currently implemented. The image quality control step requires manual inspection of cropped images to identify unusable data, followed by automated removal using a macro based on user-defined image lists. As a result, total analysis time may vary depending on the number of images analyzed and the user’s level of proficiency, making it difficult to estimate in advance. In addition, users require basic proficiency in both R and Fiji/ImageJ to successfully execute the protocol.

The radial spinning macro is computationally demanding. In this paper, the protocol was run efficiently on a computer equipped with a 12^th^ Gen Intel® Core™ i7-12700H processor (2.30 GHz), 32 GB RAM, and Windows 11. Performance on systems with lower computational capacity has not been systematically evaluated and is therefore not addressed in this protocol.

Lastly, the protocol relies on a predefined image file-naming convention for the analysis scripts and macros to function correctly. Any adjustments to the image nomenclature must also be implemented in the corresponding ImageJ macros and R scripts.

## Troubleshooting

### Problem 1

Failed image processing due to missing or incorrect spatial calibration (related to step 3–9).

The background subtraction macro and all subsequent analysis steps require images to be spatially calibrated in microns (μm/pixel). If images lack calibration or contain incorrect scaling metadata, macros that depend on spatial measurements will fail or produce inaccurate results.

### Potential solution

During image acquisition, ensure that spatial calibration is enabled in microscope software and that exported image files retain metadata containing pixel size information.

For batch correction of unscaled images, use the macro “Macro for scaling multiple images.” Before running the macro, replace the placeholder values (“x”) on line 16 for Distance in pixels and Known distance with the correct values obtained from the metadata. The macro will prompt for selection of an input directory containing unscaled images and will generate an output folder (“Scaled”) containing spatially calibrated images.

### Problem 2

Output failure due to file names not being recognized by the analysis code (related to step 3–9).

ImageJ macros and R scripts rely on consistent image file naming to correctly identify channel identifiers, time points, and other metadata.

### Potential solution

When modifying image file names, ensure that the naming convention described in this protocol is followed exactly, including capitalization, spacing, and special characters. Deviations from the prescribed naming scheme can prevent the analysis code from correctly locating or parsing input files, resulting in missing or failed outputs. Image naming during acquisition may be cumbersome, particularly when microscope acquisition software does not support batch naming. Use external batch-renaming tools (e.g., Bulk Rename Utility) prior to analysis.

### Problem 3

Excessive or inadequate background subtraction (related to step 3–9).

The background subtraction macro applies a rolling-ball radius of 50 pixels to all images acquired from different imaging channels. This value is optimal for the images generated in this protocol but may not be optimal for all TIRF microscopy immune synapse images, as signal intensity, background level, and feature size can vary between channels.

### Potential solution

Use a smaller rolling-ball radius to remove background at finer spatial scales. However, this may also remove dim or small true signals. Conversely, apply a larger radius to remove broader background variations. Note that a large radius can partially subtract real fluorescent features if set too large. Optimize the rolling-ball radius using a subset of representative images from each channel to maximize the signal-to-noise ratio and apply these channel-specific values consistently across the dataset using the macro.

### Problem 4

Incorrect or inconsistent base/parent directory (related to step 13, 20, 31).

### Potential solution

Define a single working directory on the computer in which all raw TIRF images are stored prior to analysis. When specifying input and output directories in R, locate this parent folder, copy its full directory path, and paste it consistently into the relevant sections of the code. Use this parent directory for all subsequent analyses, adding subdirectory paths only when required for specific analysis steps.

### Problem 5

Loss of synapses during center-based cropping (related to step 19–22).

The center-based cropping step in the R script excludes synapses located near the image edges, as these do not allow extraction of a 340 × 340-pixel square centered on the synapse. Depending on the acquisition strategy and the number of synapses within 340 pixels of the image boundary, this can result in substantial loss of samples for fluorescence measurements.

### Potential solution

As an alternative, measure intensity profiles using the initial (400 × 400 pixel) crops without center-based cropping by applying the corresponding ROIs. Note that radial averaging and image rotation (“spinning”) cannot be applied to these images, as these analyses require centered synapses.

To minimize this issue, during acquisition, the stage can be adjusted to avoid positioning features of interest near the image edges.

## Resource availability

### Lead contact

Further information and requests for resources and reagents should be directed to and will be fulfilled by the lead contact, Lena Svensson (lena.svensson@umu.se).

### Technical contact

Technical questions on executing this protocol should be directed to and will be answered by the technical contact, Lewis Murugu (lewis.murugu@umu.se).

### Materials availability

This study did not generate new unique reagents.

### Data and code availability

All related code and raw and processed images used in this protocol are available in an open access deposition at Zenodo (https://doi.org/10.5281/zenodo.17913303).

## Acknowledgments

This work was supported by the 10.13039/501100002794Swedish Cancer Society grants CAN2018/696 and 21 1613 (L.S.), 10.13039/501100004359Swedish Research Council grant 2018-05229 (L.S.), 10.13039/100016756Kempe Foundation SMK-2060 (L.S.), and grants from the Sandströms Foundation, Bäckströms Foundation, Insamlingsstiftelserna, and Umeå University (L.S.). J.H. is supported by the 10.13039/501100004359Swedish Research Council grants 2024-03952 and 2024-06085, the 10.13039/501100002794Swedish Cancer Society grant 23 3102 Pj, and the 10.13039/501100001729Swedish Foundation for Strategic Research ITM24-0035.

## Author contributions

Conceptualization, L.M. and L.S.; investigation, L.M. and H.P.; code, L.M. and J.H.; writing – original draft, L.M.; writing – review and editing, L.M., J.H., H.P., M.B., and L.S.; resources, M.B. and L.S.; funding acquisition, L.S.

## Declaration of interests

The authors declare no competing interests.
